# Anthrax outbreak surveillance and response in Arba Minch, Ethiopia: After-action review

**DOI:** 10.4102/jphia.v16i1.1293

**Published:** 2025-08-07

**Authors:** Yaregal Fufa, Tsion A. Desalegn, Negash Abera, Abel Getu, Yonas A. Tufa, Yeshambel W. Demlie, Ammar Barba, Mesgana Befekadu, Moti Edosa, Anteneh D. Aliyu, Zerihun D. Doffana, Ermias Wolde, Yoseph Nigussie Feleke, Melkamu M. Mengesha, Dessalegn Ajema, Muluneh G. Garedew, Temesgen Kabeta Chala, Tolera H. Wakjira, Tigist Belete, Fekadu Adugna, Nebiyu Dereje, Raji Tajudeen, Melkamu Abte

**Affiliations:** 1 Center of Public Health Emergency Management, Ethiopian Public Health Institute, Addis Ababa, Ethiopia; 2 Africa Centers for Disease Control and Prevention, Addis Ababa, Ethiopia; 3 Departments of Population and Family Health, Jimma University, Jimma, Ethiopia; 4 Department of Environmental Anthropology, Development and Conservation, Ethiopian Civil Service University, Addis Ababa, Ethiopia; 5 Department of Knowledge Translation, Ethiopian Public Health Institute, Addis Ababa, Ethiopia; 6 School of Public Health, Arba Minch University, Arba Minch, Ethiopia; 7 Department of Health Policy and Management, Jimma University, Jimma, Ethiopia; 8 EPR Cluster, World Health Organization, Addis Ababa, Ethiopia

**Keywords:** Anthrax outbreak, surveillance, response, preparedness, after-action review, outbreak response, public health

## Abstract

**Background:**

Anthrax is a serious infectious disease affecting animals and humans and remains a public health issue in developing countries.

**Aim:**

This study assessed the overall anthrax outbreak response and identified strengths, challenges, and best practices during surveillance, preparedness, and response in Ethiopia from May 2022 to July 2022.

**Setting:**

Conducted in Arba Minch Town, Gamo Zone, Southern Ethiopia.

**Methods:**

An after-action review (AAR) using qualitative methods was performed. Experts included clinicians, public health professionals, and government officials were involved in the outbreak response. Data were collected via focus group discussions, key informant interviews, and observations, recorded digitally. Thematic analysis was used.

**Results:**

Strong stakeholder engagement and coordination were evident, with mass vaccinations aiding control. However, gaps included unprepared logistics, poor communication, and insufficient training. Despite anthrax being immediately notifiable, weaknesses in surveillance detection and reporting were found. Coordination was effective with intersectoral collaboration and commitment, leading to a commendable, lifesaving response once initiated.

**Conclusion:**

The study revealed effective engagement and coordination but identified gaps in preparedness and communication. Addressing these through capacity-building and stronger preparedness is crucial for future outbreak management.

**Contribution:**

This study provides valuable evidence-based insights to improve health preparedness and response in the African context.

## Introduction

Anthrax is a serious disease usually caused by *Bacillus anthracis*, a Gram-positive, spore-forming, rod-shaped bacterium.^1^ People usually typically get sick with anthrax if they come into contact with infected animals or contaminated animal products.^2^ The bacteria produce extremely potent toxins, resulting in high morbidity and mortality rates.^1^

Anthrax in humans is often a result of contact with infected meat from livestock and wildlife.^1^ Cutaneous anthrax is the most common form in humans, while inhalation and gastrointestinal forms occur less frequently.^1^ Cutaneous anthrax accounts for up to 95% of cases and presents as itchy papules or vesicles on the skin, typically on the face, neck, forearms and hands. Within 7–10 days, these lesions can develop into skin ulcers.^3^ Annually, there are between 2000 and 20 000 reported human cases of anthrax worldwide.^4^ Anthrax remains endemic in many countries across sub-Saharan Africa, Asia, southern Europe, the Americas and certain areas of Australia.^4^ Throughout sub-Saharan Africa, anthrax is endemic to certain areas. Within these endemic areas, it usually breaks out during the rainy season, which helps the bacteria survive in the soil.^5^ Even though the ongoing presence of the disease in wildlife across various parks, South Africa typically reports less than five and occasionally zero human outbreaks annually.^6^ A study in 2018 stated that despite having successful control programmes in Botswana, Zimbabwe and Zambia, the disease remained endemic in at least the latter two countries.^7^

Anthrax has become a disease of public health and economic importance because of its increased incidence both in humans and animals and also impairs the livelihood of human beings.^8^ In Ethiopia, on 24 May 2021, an anthrax outbreak was discovered in the Southern Nations, Nationalities and Peoples Region (SNNPR), Gamo Zone, Arba Minch Town. The first case was discovered at the general hospital in Arba Minch on 04 June 2021. The Public Health Emergency Operations Center (PHEOC) at the Zonal Health Office was alerted to lead the response to public health emergencies.

Following the outbreak and its response, after-action review (AAR) was done with the objective of reviewing lessons learned from anthrax outbreak management in Gamo Zone, Arba Minch Town. Therefore, the aim of this qualitative review is to identify best practices, gaps, lessons learned and how these practices can be maintained, improved, institutionalised and shared with relevant stakeholders following an emergency response to a public health event.

## Research methods and design

### Study design

The present study employed a qualitative research approach with thematic analysis. The purposive sampling method was used.

### Study setting

This study was conducted in Arba Minch Town, Gamo Zone, Southern Ethiopia. Arba Minch Town is located approximately 441 km south of the capital town Addis Ababa. The average temperature in the area ranges from 16 °C to 33 °C, creating favourable conditions for agriculture and livestock rearing. It has a total projected population of 120 736 (Male 60 127 : Female 60 609) at the time of the occurrence of the outbreak.^9^

### Study population

The study included purposively selected experts such as clinicians, public health professionals and government officials who were directly engaged in anthrax outbreak response activities.

### Data collection and sampling strategy

Data were collected through face-to-face focus group discussions (FGDs), in-depth interviews and observation. Data collection tools, an interview guide and a semi-structured checklist were prepared to generate data from rapid response team (RRT) members, health professionals and selected community members.

A total of four FGDs were conducted: two FGDs for health centre RRT members; one for health professionals comprising clinicians, pharmacists, pharmacy technicians, and dermatologists and one for selected community members. The FGD session involved 6–8 participants. Conversations were recorded using a digital audio voice recorder. Before each FGD, an introduction, ground rules and the study’s objective were discussed. A total of 27 men and 13 women participated in the discussion, and there were no refusals during any of the FGD sessions. Focus group discussions took place, lasting between an average time of 60 min to 120 min. Group sizes varied from 6 to 8 participants. Two FGDs occurred at the community level while the remaining two were conducted at health facilities and the national level in the workplace.

For individual interviews, eight key informants and experts engaged in the anthrax outbreak response activities from woreda (districts) to federal level. Individual informants included woreda health office heads, zonal public health emergency management (PHEM) unit, regional PHEM leads, national PHEM unit and purposively selected RRT members at each level based on their active participation and experience in outbreak response activities. Interviews and discussions were conducted on questions deriving from the core AAR functional areas, indicators and themes: surveillance, response and coordination.

#### Observation

During the review, several key indices were observed: response time, collaboration among RRT members, community engagement, resource availability and communication clarity. Response time measured how quickly the team acted after the outbreak notification. Collaboration assessed the teamwork among health professionals and community leaders. Community engagement looked at local involvement in response efforts, while resource availability focused on access to medical supplies and personnel. Communication clarity evaluated how effectively the community was informed. Each index was monitored over two weeks following the outbreak to assess the overall effectiveness of the health interventions.

### Data management and analysis

The data were recorded using a digital voice recorder. Translation from Amharic to English language and transcription, management and analysis were done using MAXQDA version 2020. The data were content coded for thematic analysis. Initial coding activities were based on prior conceptual categories, and further coding concepts were derived from the data. MAXQDA allowed for a detailed examination of the coded data, making it easy to identify common themes and understand the relationships between different codes. This thorough analysis led to the identification of three main themes, each providing important insights into the patterns and narratives found in the data.

After presenting the results, we will discuss the key issues derived from the findings, placing them within the context of national and international standards and relevant protocols. We will also compare these findings with similar reports from other settings. The result and discussion will follow a thematic scope approach, beginning with preparedness, coordination, response and surveillance. Finally, conclusions will be drawn based on the key results and discussions.

### Ethical considerations

This study received ethical approval from the Ethiopia Public Health Institution, with approval reference number ethical codes clearance approved by the Ethiopian Public Health Institute (EPHI), ref: 64/683. Informed consent was obtained from all participants before data collection. Participants were informed about the study’s purpose, their voluntary participation, the right to withdraw at any time and measures taken to ensure confidentiality. Personal identifiers were removed to protect participant anonymity, and all data were stored securely with access limited to the research team.

According to the Federal Negarit Gazeta of Federal Democratic Republic of Ethiopia (FDRE) Regulation No. 301-2013 EPHI establishment council of the Ministry of Regulation page, 7175, 20th year 10 Addis Ababa 1st January 2014, the Ethiopia Public Health Institute has the power and duties to conduct during epidemics or any other public health emergency or public health risk, on-site investigation when deemed necessary, verify outbreaks, issue alert, provide warning and disseminate information to the concerned organs, mobilise or cause the mobilisation of resources and support the response activities carried out at woredas (district), zones and regional levels as deemed necessary.

## Results

### Preparedness

Preparedness for anthrax outbreaks was generally described as weak in terms of documentation of key tools that map and guide preparedness, budget, trained human resources, PHEM training and cascading and laboratory capacity at lower levels. However, there are efforts that can be mentioned as a strength, including the availability of a well-organised laboratory at the national level, to detect anthrax. Limitations regarding training were a lack of cascading to lower levels, particularly at the health facility levels. Concerns regarding dedicated staff for PHEM activities in the health system structure, availability of resources and preparedness are summarised in the following quote:

‘… PHEM structure and resources are insufficient. No dedicated budget for outbreak response before its occurrence, implying a lack of preparedness and emergency medicine and supplies.’ (KII from Zonal PHEM Unit, 2021)

The lack of documented plans and supplies for anthrax was not available, as noted by a Woreda health office PHEM officer:

‘As anthrax had not happened previously in our area, we did not consider it as a potential threat. Consequently, we did not plan for it. Plans for less important threats would need more resources.’ (KII from Woreda PHEM officer, 2021)

A summary of details for each of the themes is presented in Table 1.

**TABLE 1:** Summary of key findings, challenges, and strengths by theme from the after-action review.

Theme	Key findings	Challenges	Strengths
Preparedness	No documentation of VRAM and EPRP and did not categorise hotspot areas based on anthrax vulnerability. There was no backup plan, task force or surge capacity. Limited capacity-building training and lack of cascading of trainings specific to anthrax. Anthrax has not occurred in the area for the past 50 years; as a result, there were no prepositioned supplies targeting anthrax. Anthrax was not considered as a potential threat in the area.	Lack of budget allocation for preparedness, particularly at lower levels. There was no preparation to mitigate the anthrax outbreak. Some of the PHEM focal points received short-term orientation during the outbreak but not basic PHEM training. Lack of anthrax guidelines and anthrax-specific training prior to the outbreak.	Availability of PHEM guideline. Mobile vans and mass media were used for community awareness creation. Well-equipped laboratory and skilled manpower at the national level. Digital communication channel via the Telegram application.
Coordination	The regional health office led the coordination, and a regional EOC was established. Technical working group (TWG) was established and deployed to the outbreak area. Coordination was evident in various areas, including social mobilisation, surveillance, preventive activities, monitoring and feedback, logistics, supply delivery and collaboration with partner organisations.	There was no clearly documented TOR or MOU at the operational level to identify roles and responsibilities and hence accountability. At the regional level, there was no dedicated task force for anthrax. Failure to engage community leaders in the response.	Conducting coordination meetings for updates, feedback and important decisions. All relevant stakeholders were mapped and engaged in the response. The One Health approach.
Surveillance and laboratory	The detection has come so late after the occurrence of the first incident cases in the town. Poor surveillance system at a lower level on early detection of the outbreak index case. Improved laboratory capability to detect anthrax serologically and using the PCR after the outbreak.	Resource scarcity, particularly computers for epidemiological data management and utilisation to guide the response. Inability to identify the source of the outbreak early challenged the response activity. Late communication of test results and lack of laboratory capacity both at the zonal and regional levels.	Use of digital technology to communicate information between field staff and offices. Prompt provision of orientation to public and private health professionals. House-to-house case-finding effort and linking this to mass treatment at schools and working with regulatory bodies.
Response, risk communication and community engagement	The RRT included health professionals and veterinary professionals and risk communication experts from different organisations. A close follow-up from the Zonal Health department with frequent field visits. Information about the outbreak was available to the community in different platforms in places where people congregate (awareness was created).	Lack of a specific budget for risk communication. Poor health-seeking behaviour of the community. Delay in case management, shortage of drugs and supplies. Community was unaware of the signs and symptoms of anthrax. Raw meat being the usual and favoured diet.	Anthrax treatment guidelines and case definitions were immediately prepared. Mobilisation of human resources. Presence of dermatologists in the RRT. Arba Minch University allocated a budget to train health extension workers.

EOC, emergency operation centre; TOR, terms of reference; RRT, rapid response team; VRAM, vulnerability assessment and risk mapping; EPRP, emergency preparedness and response plan; PHEM, public health emergency management; MOU; memorandum of understanding; PCR, polymerase chain reaction.

### Coordination

During the outbreak of anthrax, coordination was made at different levels: at national, regional, zonal and community levels after the detection and confirmation of cases. Regarding coordination at the federal level, a senior officer at the Ethiopian Public Health Institute (EPHI) noted that the ‘TWG were established and the team also was deployed to the outbreak area immediately, Gamo Zone, Arba Minch Town’ (EPHI KII, 2021). He further added that ‘The RRT team were activated and met regularly with staff from different stakeholders who engaged in the outbreak management’ (EPHI KII, 2021). The team coordinated the daily activities: logistics supply, maintenance, community mobilising and finance. One of the study participants from Gamo Zone also stated that ‘the coordination is well-organised and it includes several stakeholders from governmental and non-governmental organisations’ (Gamo Zone Health Department KII, 2021).

Regarding regular coordination meetings, key informants from various sectors claimed that there was a coordination meeting with meeting minutes being documented. However, meetings were no longer held on a regular basis. In addition, lack of Terms of Reference (TOR)/memorandum of understanding (MOU) for the outbreak coordination led to overlapping of tasks and lack of ownership of the response activities.

One Health approach to anthrax outbreak response coordination was used, and One Health team was established in Gamo Zone a year before the current outbreak is occurred. KI from Gamo Zone noted that the:

‘The One Health approach significantly contributed to coordinating the response during the anthrax outbreak by promoting collaboration among various sectors, including human health, animal health, and environmental health. This integrated approach facilitated effective communication and resource sharing among public health officials, veterinarians, and environmental experts, allowing for a comprehensive understanding of the outbreak’s dynamics.’ (KII from Zonal PHEM Unit, 2021)

He further explained that: ‘The occurrence of the anthrax outbreak helped to work closely by creating a platform to exchange information on regular bases’. (KII from Zonal PHEM Unit, 2021)

See Table 1 for a summary of details related to coordination and other themes related to anthrax in Gamo Zone, Arba Minch.

### Surveillance

Surveillance involves collecting, analysing and interpreting information related to public health. An effective surveillance system enables health officials and experts to promptly detect disease outbreaks and other health events. In Ethiopia, both immediately and weekly reportable diseases are monitored, with anthrax being among the immediately reportable conditions.

The anthrax outbreak that occurred in Gamo Zone, Arba Minch Town, was first detected in Arba Minch General Hospital and reported to the Zonal Health Department on 24 May 2021; this has also been reported to EPHI. However, the case detection has come one month or a month and a half later than the occurrence of the outbreak. The health expert who first detected cutaneous anthrax cases reported that:

‘I’m not certain about the exact time between the suspected anthrax cases at Abaya Campus and their detection at Arba Minch General Hospital. It could be approximately one to one and a half months after the outbreak began.’ (Health expert KII, 2021)

The weekly reports for the region and zone were over 90% complete, but the surveillance did not pick up the outbreak. The first incidents of the outbreak were neglected as reported by a key informant at the national level:

‘At first, the health office in Arba Minch was informed, but they missed the alert. As a result, we could not quickly identify the disease, possibly because it had not been seen in the area before.’ (KII Zonal PHEM officer, 2021)

## Investigation of source of the outbreak

In this outbreak, it was not possible to identify the actual source of the anthrax outbreak, except for the different possible hypotheses put forward by the response team. Given the affected population consisted mainly of schoolchildren, the first hypotheses were associated with possible exposure to anthrax spores in contaminated soil. The other hypothesis was associated with exposure to the carcass of infected animals. This hypothesis was linked to the setting where a cluster of the first incident of skin disease of unknown origin was reported. Although students on campuses of the higher education institution use cooked meat as part of their meal in the cafeteria, workers who slaughter cattle on campus may have acquired infections from infected cattle.

In interviews conducted with health centre staff, RRT members described their level of engagement as well as that of officials visiting from the Zonal Health Department as critical:

‘[*T*]he rapid response team was strongly involved in the prevention and control efforts of anthrax by doing home-to-home visits, providing health education, reporting, referral and management of affected cases.’ (KII from Zonal Health department, 2021)

See Table 1 for a summary of details related to the response and other themes related to anthrax in Gamo Zone, Arba Minch Town.

## Discussion

As outlined in the results section, the preparedness activities, including document availability, plan and supply readiness, budget and finance, supportive supervision, human resources and capacity building, fell short of standards. As a result, many participants perceived preparedness for an anthrax outbreak as inadequate. This finding indicates that the anthrax outbreak was not considered as a public health threat, and the finding aligns with a study on the Yellow Fever Outbreak Response in Ethiopia, conducted in the same region but a different zone, which also revealed inadequate preparedness.^10^ This suggests that early preparation for public health emergencies is not a standard practice in our setup, possibly because of a lack of funds and insufficient budget allocation for regular services.

According to the PHEM guideline (2021), public health emergency preparedness involves establishing logistics and funding, developing systems for protection, prevention and response, equipping public health personnel and responders with the necessary knowledge and tools and educating the public on preventive and control measures.^11^ Contrary to the guidelines, the results section revealed significant gaps in preparedness, including the lack of well-organised capacity-building training for healthcare providers at all levels and the absence of regular supportive supervision on PHEM before the outbreak. This underlines how the preparedness for an anthrax outbreak in our health system fell short of expected standards. One possible reason for these gaps could be the absence of an emergency operation centre (EOC) at the zonal level, contributing to the overall deficiencies in the preparedness pillar.

The national guidelines also emphasise coordination activities such as identifying sectors and partners, developing TOR and forming TWG or RRT. Coordination among multi-sectoral organisations was often limited. As a result, many participants perceived the preparedness for an anthrax outbreak as inadequate. This finding reveals that the anthrax outbreak was not considered a public health threat, and the finding aligns with a study on the Yellow Fever Outbreak Response in Ethiopia, conducted in the same region but a different zone, which also revealed inadequate preparedness.^10^ This suggests that early preparation for public health emergencies is not a standard practice in our setup, possibly because of a lack of funds and insufficient budget allocation for regular services.

According to the PHEM guideline (2021), public health emergency preparedness involves establishing logistics and funding, developing systems for protection, prevention and response, equipping public health personnel and responders with the necessary knowledge and tools and educating the public on preventive and control measures.^11^ Contrary to the guidelines, the results section revealed significant gaps in preparedness, including the lack of well-organised capacity-building training for healthcare providers at all levels and the absence of regular supportive supervision on PHEM before the outbreak. This underlines how the preparedness for an anthrax outbreak in our health system fell short of expected standards. One possible reason for these gaps could be the absence of an EOC at the zonal level, contributing to the overall deficiencies in the preparedness pillar.

The national guidelines also emphasise coordination activities such as identifying sectors and partners, developing TOR and forming TWG or RRT. Coordination among multi-sectoral organisations to manage public health disasters involves establishing links both horizontally and vertically.^11^ This study found that a coordination system was established with all relevant stakeholders, who had clear roles despite the absence of a TOR. Regular meetings were held, especially in the initial weeks; this approach largely aligns with the expected coordination standards outlined in the national guidelines.

One of the challenges faced was the competing priority of National Elections during the outbreak, which hindered the full engagement of political leaders in controlling the situation. Similar reports from Africa indicate that effective coordination during health service emergency management often encounters various challenges, including natural factors and socio-political and infrastructural issues.^12^

Surveillance activities were conducted following the detection and confirmation of anthrax. This included active case searches and descriptive data analysis for decision-making. However, case notification and detection were delayed by over a month because of insufficient laboratory capacity at zonal and regional levels. Despite World Health Organization (WHO) recommendations for prompt detection of immediately reportable diseases, this study identified a significant delay in reporting the first cluster of an unspecified skin disease at Abaya Campus, Arba Minch University. Similarly, a study conducted in the Amhara region of Ethiopia revealed a significant delay during the anthrax outbreak, with over 100 days passing between the initial report and the start of the outbreak investigation.^13^

The response evaluates whether appropriate measures were implemented across all intervention aspects, including case management, prevention and control and vaccination campaigns. The overall response to the anthrax outbreak began with case management and mass vaccinations. This strategy aligns with WHO guidelines, which recommend starting with treatment followed by vaccination.^14^

The response to the anthrax outbreak in Arba Minch was coordinated with the involvement of all stakeholders, from federal to local health authorities. Since the endorsement of the PHEM guideline, Ethiopia has been addressing public health threats through a collaborative approach that includes federal, regional and local levels. Similarly, after-action assessments of the response to the anthrax in the United States (US) also indicated public health functions are carried out with the participation of federal, regional and local health authorities.^15^

The anthrax response plan involved both human and animal health authorities. Bhutan’s guideline on anthrax outbreak investigation and response clearly states that the response plan should include both sectors.^16^ This alignment supports health system integration efforts in line with International Health Regulation requirements. Therefore, promoting a One Health approach to zoonotic diseases in Ethiopia is crucial. In addition, the public health emergency response emphasises rapid outbreak assessment, investigation, control and prevention measures, along with continuous monitoring. A swift and effective response can limit the number of cases, reduce the geographical spread, shorten the outbreak duration and decrease fatalities. These benefits save resources and reduce morbidity and mortality. Strengthening epidemic response, especially at the Woreda and community levels, and focusing on response strategies and ongoing evaluation are crucial.

The result of this study also indicated that the response faced shortages of drugs and supplies. This situation highlights a broader issue: public health emergency responses in sub-Saharan Africa continue to be hampered by underfunding.^17^ Case management is supported by drugs like ciprofloxacin or doxycycline and amoxicillin for paediatrics. This approach was supported by a review conducted in Ethiopia, which reveals *Bacillus anthrax* is susceptible to antibiotics.^18^ This might indicate that accessing pharmaceuticals and other supplies is still a challenge in resource-limited countries because of limited local manufacturing capacities.

Communities’ traditional health preferences have posed challenges to the public health response to the outbreak, causing delays in the detection and early identification of vulnerable individuals. Similarly, the 2001 anthrax attack in the US, known as Amerithrax, revealed that federal, regional and local health authorities faced similar challenges in their response efforts.^15^

Communicating in the local language is generally ideal for raising awareness, and it is important to identify and convey signs and symptoms through various communication channels.^19^ The study results indicated that local media, such as television and frequency modulation (FM) radio, along with the organisation’s Facebook and Telegram channels, were utilised. Additionally, risk communication and community engagement (RCCE) mobile vans, posters and health education were employed to reach a broader community.

The current study’s findings are similar to those from the Zimbabwe anthrax outbreak, where RCCE activities were carried out after the outbreak. Information, education and communication materials were not available during the early stages of the outbreak response and only became available later.^20^ For coordinating RCCE during the outbreak response, a platform was established soon after the outbreak occurred, through the task force. This approach was more effective compared to the anthrax outbreak response in Zimbabwe, where the district lacked functional zoonotic committees.^21^

This study found that the community’s response to the message varied, with some individuals resisting or not accepting it. Consequently, there was a noticeable negligence in following the recommended precautions. According to the WHO, this may be attributed to a loss of trust between the respondents and the key target audiences.^22^ Trust is one of the five principles of risk communication as outlined by the WHO.^23^

According to the WHO, one of the action steps for RCCE readiness and response is to prepare a budget.^22^ However, it was found that a budget dedicated to risk communication was not secured before the outbreak, both as a preparedness measure and during the anthrax outbreak response. Conducting effective community education on sanitation practices and food hygiene for the general public and those in the food industry is essential to ensure effective waste disposal and improve food handling practices.^11^ In the study area, after the COVID-19 pandemic, various institutions, including healthcare facilities, have incorporated IPC programmes into their structures. This already activated COVID-19 IPC system was utilised during the anthrax outbreak, with health extension workers disseminating IPC practices to the community.

## Conclusion

This assessment examined the challenges, gaps, best practices and lessons learned from outbreak management using focus group discussions, key informant interviews (KIIs), observations and document reviews. It evaluated five key dimensions of the AAR: preparedness, surveillance, coordination, risk communication and community engagement.

The surveillance system for anthrax, classified as an immediately notifiable disease, was inadequate, causing delays in reporting to the Integrated Disease Surveillance and Response (IDSR) because of insufficient knowledge among health professionals and poor communication channels. Coordination began two months after the first notification but was ultimately effective, demonstrating strong inter-sectoral collaboration. While the first case was detected late, the response was commendable and aligned with established standards, effectively saving lives.

Significant gaps included the lack of essential documents like anthrax guidelines and preparedness plans, as well as unprepared logistics for medical supplies. To address these issues, the Ethiopian Public Health Institute, the Ministry of Health and partners should enhance capacity building and strengthen preparedness.

Establishing an emergency operation centre (EOC) and an incident management system (IMS) is vital for better communication and coordination during public health emergencies. The EOC acts as a central hub for decision-making and information sharing, while the IMS helps manage incidents effectively. Together, they improve communication and coordination during public health emergencies.
